# Telocytes in neuromuscular spindles

**DOI:** 10.1111/jcmm.12015

**Published:** 2013-04-28

**Authors:** Lucio Díaz-Flores, Ricardo Gutiérrez, Francisco J Sáez, Lucio Díaz-Flores, Juan F Madrid

**Affiliations:** a Department of Anatomy, Pathology, Histology and Radiology, Faculty of Medicine, University of La LagunaTenerife, Spain; b Department of Cell Biology and Histology UFI11/44, School of Medicine and Dentistry, University of the Basque Country, UPV/EHULeioa, Spain; c Department of Cell Biology and Histology, School of Medicine, Regional Campus of International Excellence, “Campus Mare Nostrum”, University of MurciaEspinardo, Spain

**Keywords:** Telocytes, muscle spindles, stromal cells, telopodes, musculoaponeurotic fibromatosis, inflammatory myopathy

## Abstract

A new cell type named telocyte (TC) has recently been identified in various stromal tissues, including skeletal muscle interstitium. The aim of this study was to investigate by means of light (conventional and immunohistochemical procedures) and electron microscopy the presence of TCs in adult human neuromuscular spindles (NMSs) and lay the foundations for future research on their behaviour during human foetal development and in skeletal muscle pathology. A large number of TCs were observed in NMSs and were characterized ultrastructurally by very long, initially thin, moniliform prolongations (telopodes – Tps), in which thin segments (podomeres) alternated with dilations (podoms). TCs formed the innermost and (partially) the outermost layers of the external NMS capsule and the entire NMS internal capsule. In the latter, the Tps were organized in a dense network, which surrounded intrafusal striated muscle cells, nerve fibres and vessels, suggesting a passive and active role in controlling NMS activity, including their participation in cell-to-cell signalling. Immunohistochemically, TCs expressed vimentin, CD34 and occasionally c-kit/CD117. In human foetus (22–23 weeks of gestational age), TCs and perineural cells formed a sheath, serving as an interconnection guide for the intrafusal structures. In pathological conditions, the number of CD34-positive TCs increased in residual NMSs between infiltrative musculoaponeurotic fibromatosis and varied in NMSs surrounded by lymphocytic infiltrate in inflammatory myopathy. We conclude that TCs are numerous in NMSs (where striated muscle cells, nerves and vessels converge), which provide an ideal microanatomic structure for TC study.

## Introduction

A peculiar type of interstitial (stromal) cells named telocytes (TCs; cell bearing long prolongations/stromal cells with telopodes – Tps -) 1 has been described and adopted by several laboratories [for review see 1, 2 and for details visit http://www.telocytes.com]. Formerly called interstitial-like (Cajal-like) cells, TCs have been observed in several tissues and organs, including skin, respiratory tract, cardiovascular system, gastrointestinal tract, liver, gallbladder, pancreas, parotid gland, female reproductive system, breast, placenta, urinary tract and meninges and choroid plexus 3–25.

In skeletal muscle interstitium, TCs have recently been identified close to myocytes, satellite cells, nerve endings and capillaries, suggesting a role in muscle regeneration and repair, and in intercellular signalling 26–28. In this way, it is of interest to investigate the presence of TCs in neuromuscular spindles (NMSs), intramuscular microanatomic structures related to muscle tone. This work was therefore undertaken to search for TCs in NMSs of normal human striated muscle and lay the groundwork for future studies on TCs in NMSs during foetal development and pathological conditions of striated muscle, including inflammation and tumour invasion of the muscle.

## Material and Methods

### Tissue samples

The archives of the Department of Anatomical Pathology of the University Hospital of the Canary Islands were searched for histopathological specimens containing areas with normal skeletal muscle of random position in the muscular system. In specimens embedded in paraffin, histological serial sections were again made and 16 NMSs were observed from these areas. The specimens were studied by means of conventional and immunohistochemical procedures. In specimens processed for electron microscopy, four new NMSs were obtained. Six NMSs were also studied from specimens, showing skeletal muscle with histological modifications (in two cases of musculoaponeurotic fibromatosis and in one case of inflammatory myopathy). Five NMSs were examined in skeletal muscle obtained from foetal autopsy (22–23 weeks of gestational age estimated from the menstrual cycle and the last menstrual period). NMSs from pathological-modified skeletal muscle and foetus were only processed for light microscopy studies (conventional and immunohistochemical). All protocols were performed in accordance with international ethical guidelines.

### Histology and immunohistochemistry

For light microscopy, specimens were fixed in a buffered neutral 4% formaldehyde solution, embedded in paraffin and cut into 4-μm thick sections, which were stained with haematoxylin & eosin and PAS-Alcian Blue.

For immunohistochemical procedures, in all the specimens with NMSs under conventional light microscopy, 3-μm thick sections were cut and attached to silanized slides. After pre-treatment for enhancement of labelling [antigen retrieval PT-Link (Dako, Glostrup, Denmark), Ref. 1012], the sections were blocked with 3% hydrogen peroxide and then incubated with primary antibodies (10–40 min.).

The primary antibodies (Dako) used in this study were CD34 (dilution 1:50), code no. M-7168; CD31 (dilution 1:50), code no. M-0823; CD117 (c-kit; dilution 1:50), code no. A-4502; α-smooth muscle actin (SMA; dilution 1:50), code no. M-0851; desmin (dilution 1:50), code no. M-0760; h-caldesmon (dilution 1:50), code no. M-3557; vimentin (dilution 1:100), code no. M-0725; S-100 protein (dilution 1:100), code no. H-0066; epithelial membrane antigen (EMA; dilution 1:100), code no. M-0613; cytokeratin (CK) AE1/AE3 (dilution 1:100), code no. N-1590; neurofilament protein (dilution 1:50), code no. M-0762; collagen IV (dilution 1:50), code no. M-0785 and laminin (dilution 1:20), code no. M-0638. The immunoreaction was developed in a solution of diaminobenzidine and the sections were then briefly counterstained with hematoxylin, dehydrated in ethanol series, cleared in xylene and mounted in Eukitt®. Positive and negative controls were used.

### Transmission electron microscopy

For electron microscopy, specimens were fixed in a glutaraldehyde solution, diluted in 2% with sodium cacodylate buffer, pH 7.4 for 6 hrs at 4°C, washed in the same buffer, post-fixed for 2 hrs in 1% osmium tetroxide, dehydrated through a graded acetone series and embedded in epoxy resin. Semi-thin sections (1.5 μm) were mounted on acid-cleaned slides, and stained with 1% Toluidine blue. Ultrathin sections were double stained with uranyl acetate and lead citrate. The grids were examined at 60 kV with a JEOL 100B electron microscope.

## Results

### Cell components other than TCs in normal NMSs

**Fig. 1:**
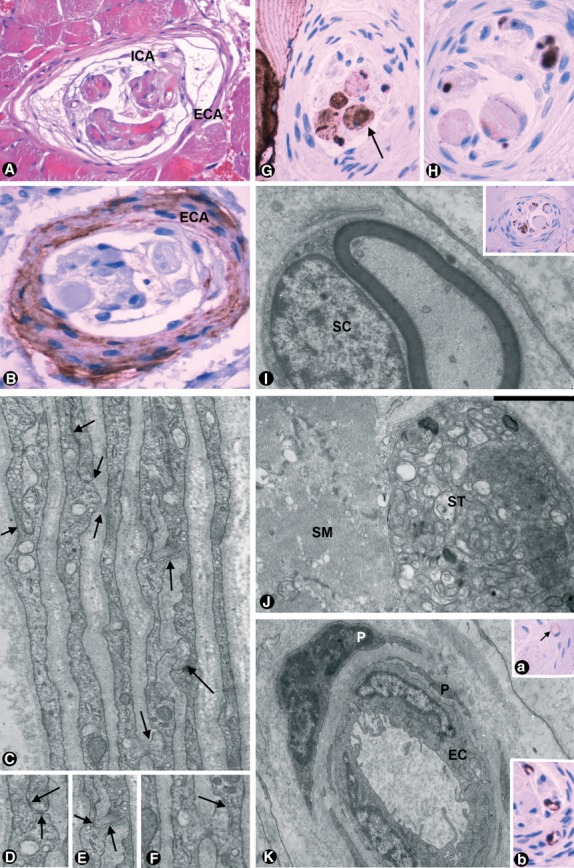
Cell components other than telocytes in normal NMSs. (**A**) An NMS, with external (ECA) and internal capsule (ICA), in routine H&E study (×220). (**B**) EMA-positive perineural cells in external capsule (×340). (**C–F**) Ultrastructural characteristics of perineural cells with presence of basement membrane, pinocytotic vesicles and junctions (arrows; C, ×9000; D–F, ×15,000). (**G**) Desmin-positive intrafusal striated muscle fibres (×300). (**H**) Positivity for anti-neurofilament protein in the axons of nerve intrafusal fibres (×300). (**I**) Typical ultrastructure of Schwann cells (SC; ×12,000). Insert: positivity in these cells for anti-S-100 protein (×220). (**J**) Sensory terminals (ST), with densely packed mitochondria, adjacent to a skeletal muscle cell (SM; ×20,000). (**K**) Intrafusal capillary with endothelial cells (EC) and pericytes (P; ×7500). Insert 1 Ka, positivity of the endothelial cells for anti-CD31 (arrow; ×220). Insert 1 Kb, expression of α-SMA in pericytes (×340).

Normal human NMSs (Fig. 1A), with both external and internal capsules, generally contained two to five striated muscle fibres (some muscle spindles had up to 10 intrafusal muscle fibres), sensory and motor nerve fibres and nerve terminals, and capillary vessels. Thus, the cells other than TCs in normal NMSs included perineural cells of the multi-layered external capsule, skeletal muscle fibres, Schwann cells around nerve fibres and vascular cells (endothelial cells and pericytes).

The perineural cells, which made up most of the external capsule (on occasion resembling the capsule of Paccinian corpuscles with concentric, loosely arranged lamella), expressed EMA immunohistochemically (Fig. 1B) and, under electron microscopy, the presence of a basement membrane, pinocytotic vesicles and junctions with other perineural cells, in the same or adjacent layer (Fig. 1C–F).

The intrafusal striated muscle fibres, including the two major types: ‘nuclear chain fibres’ (smaller diameter, single axial chain of nuclei, attached at their poles to the capsule or to the sheaths of nuclear bag fibres) and ‘nuclear bag fibres’ (greater in diameter, spindle fibres with an equatorial area of numerous small nuclei—nuclear bag of Barker—extending beyond the surrounding capsule to the endomysium of nearby extrafusal muscle fibres) were easily identified under light microscopy by their typical morphology and desmin immunopositivity (Fig. 1G).

The nerve intrafusal fibres showed immunohistochemical positivity for anti-neurofilament protein in their axons (Fig. 1H), S-100 expression in Schwann cells (Fig. 1I insert) and, ultrastructurally, a typical basement membrane around Schwann cells (Fig. 1I).

Sensory nerve terminals (Fig. 1J), mainly occupied by densely packed mitochondria and few clear vesicles, were located adjacent to the skeletal muscle cells, externally surrounded by a common basement membrane and separated by an empty, narrow synaptic cleft, except where they formed dense plaques.

In the capillaries, the continuous endothelium was heavily immunostained with anti-CD34 and anti-CD31. The latter only stained endothelial cells in NMSs (Fig. 1K, insert a) and the pericytes were reactive for anti-α-smooth muscle actin (Fig. 1K, insert b). Ultrastructurally, the pericytes and endothelial cells showed typical characteristics (Fig. 1K).

### TCs in normal NMSs

**Fig. 2:**
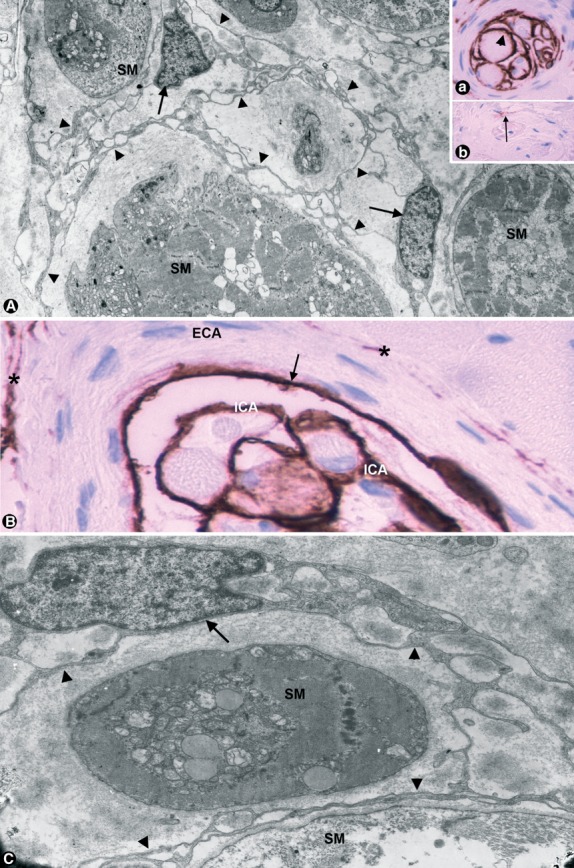
Telocytes (TCs—arrows) in normal NMSs. (**A**) Long, slender cytoplasmic processes (Telopodes: Tps—arrowheads) of TCs form a labyrinthine system surrounding other intrafusal structures. Striated muscle: SM (×5500). Insert 2 Aa: Expression of CD34 in TCs (arrowhead; ×220). Insert 2 Ab: Occasional positivity for anti-CD117 in a cell (arrow). (**B**) Detail of CD34-positive TCs forming the innermost layer (arrow) and part of the outermost layer (asterisks) of the external NMS capsule (ECA) and the internal capsule (ICA; ×640). (**C**) Ultrastructural detail of the body of a TC (arrow) and Tps (arrowheads) surrounding a striated muscle cell (SM; ×10,500).

**Fig. 3:**
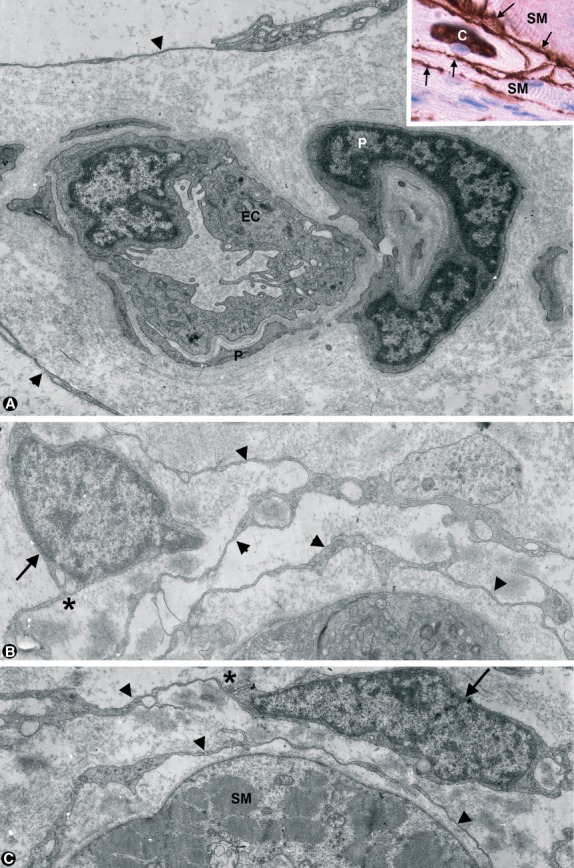
Telocytes (TCs—arrows) in normal NMs. (**A**) Telopodes (Tps—arrowheads) surrounding an intrafusal capillary, with endothelial cells (EC) and pericytes (P; ×9500). Insert: CD34-positive TCs (arrows) surrounding a capillary (C) and striated muscle cells (SM). Capillary endothelial cells are also heavily stained by CD34 (×420). (**B** and **C**) TC bodies (arrows) with triangular (B) or spindle (C) morphology and typically thin Tps (arrowheads) emerging from the cell body (asterisks), with ramifications, podomeres and podoms (×9500).

Telocytes expressing vimentin and CD34, and with characteristic ultrastructural findings, were an important component of NMSs (Fig. 2A and insert a). Only occasionally did they express c-kit/CD117 (Fig. 2A, insert b). Most TCs were located in the intrafusal connective tissue forming the NMS internal capsule (Fig. 2A and a insert). They also contributed to the external NMS capsule (Fig. 2A insert a and B), specifically making up its innermost layer and part of its outermost layer (Fig. 2B). TCs showed a slender body and two to five long, slender cytoplasmic processes (telopodes—Tps), which sometimes overlapped, forming a labyrinthine system (Fig. 2A and C). Tps were organized in a network surrounding the striated muscle fibres (Fig. 2A–C), vessels (Fig. 3A and 3A insert) and nerve fibres (Fig 1I). Each intrafusal structure was individually (partially or totally) encircled by the Tp network. The TCs of the innermost and outermost layer of the external capsule showed spindle morphology, delimiting the periaxial (subcapsular) space and incompletely separating the extrafusal regions respectively (Fig. 2B).

**Fig. 4:**
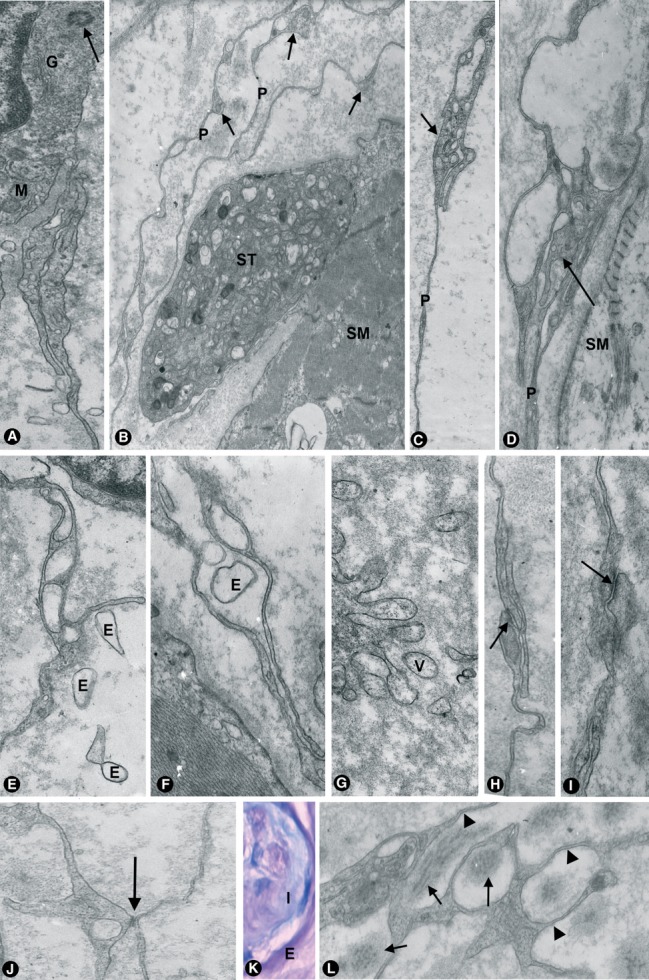
Details of cytoplasmic body and Tps, and extracellular matrix in normal NMSs. (**A**) A centriole (arrow), a small Golgi apparatus (G) and few mitochondria (M) are observed in the scarce somatic cytoplasm of a TC (×15,000). (**B–D**) Pomoderes (P) and podoms (arrows) in Tps. Striated muscle: SM. Sensory terminal: ST (×10,000). (**E–G**) Exosomes (E) and shed vesicles (V) in vicinity of Tps. Some shed vesicles are observed being released from a Tp (G). (E and F: ×12,000; G: ×15,000). (**H–J**) Junctions between Tps (arrows; ×12,000). (**K**) PAS positivity of the extracellular matrix in the NMS external capsule (E) and Alcian Blue positivity in intrafusal area (I) PAS-Alcian (×380). (**L**) Collagen fibrils (arrows) in folds of Tps (arrowheads), which show a honeycomb-like structure (×12,000).

The cellular body or nuclear region of TCs in the intrafusal connective tissue showed either triangular or spindle morphology (Fig. 3B and C). Relatively large in relation to the small content of cytoplasm in the cell body, the nucleus was ovoid and showed patches of heterochromatin above all in close proximity to the nuclear membrane (Fig. 3B and C). The thin layer of somatic cytoplasm contained polysomes, scarce cisternae of rough and smooth endoplasmic reticulum, few mitochondria, a small Golgi apparatus and centrioles (Fig. 4A). Only Tps projecting from the small cell body with initially thin processes were observed (Figs. 3B and C). Tps of moniliform aspect and with bifurcations showed extremely thin segments (podomeres) and dilated portions (podoms; Figs 3B, C and 4B–D). The podomeres contained microfilaments and ribosomes. Mitochondria, endoplasmic reticulum and some caveolae were observed in the podoms. TCs usually presented plasmalemmal caveolae. Shed vesicles and exosomes were also observed being released from and in the vicinity of Tps (Fig. 4E–G). Junctions were present between Tps (Fig. 4H–J). The TCs had no basement membrane.

### Extracellular matrix in normal NMSs

The extracellular matrix was stained by PAS in the capsule and mainly by Alcian Blue in intrafusal areas (periaxial or subcapsular space and beneath the internal capsule; Fig. 4K). Thin collagen fibrils were also observed, mainly in folds of Tps showing a honeycomb-like structure (Fig 4L).

### TCs during development of human NMSs

**Fig. 5:**
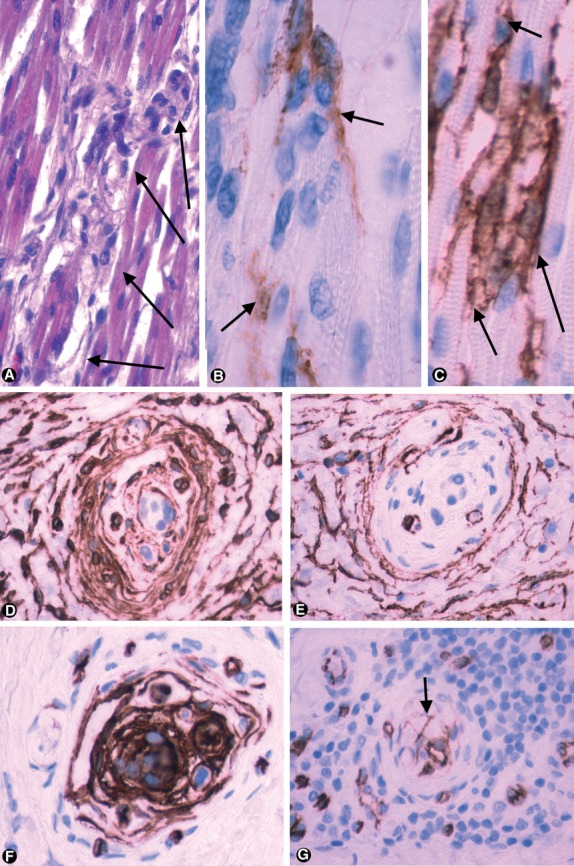
TCs during development and in muscle pathology. (**A**) Early stage of NMS formation, in routine H&E study (arrows; ×220). (**B** and **C**) Immunohistochemical expression of EMA and CD34 in developing NMs (B) a primitive and incomplete external capsule is observed from extension of EMA-positive perineural cells (arrows). (C) CD34-positive TCs, forming an internal capsule (arrows; ×640). (**D**–**F**) Residual NMSs in musculoaponeurotic fibromatosis with infiltrative muscular pattern. (D) Vimentin expression (E) actin expression. (F) CD34-positive cells, forming most of the intrafusal components (×300). (**G**) Presence of a lymphocyte infiltrate around an NMS in inflammatory myopathy. Fewer CD34-positive intrafusal TCs (arrow) (×300).

At 22–23 weeks of gestational age, early stages of NMS formation were observed (Fig. 5A). In these structures, a primitive and incomplete external capsule from extensions of perineural cells was present (Fig. 5B). Simultaneously, CD34-positive TCs increased in number compared with those in the adjacent muscle and formed a sheath surrounding primary intrafusal myotubes (Fig. 5C) and nerves (primitive internal capsule and innermost layer of the external capsule). CD34 and EMA expression for TCs and perineural cells, respectively, were good markers for identifying NMSs in early stages of formation.

### NMSs and their TCs in muscle pathology

In the two cases of musculoaponeurotic fibromatosis with an infiltrative intramuscular pattern of growth (almost complete replacement of muscle), residual NMSs were observed. The latter appeared embedded within the lesion, which was composed of proliferating myofibroblasts (vimentin and α-smooth muscle actin positivity; Fig. 5D and E) and intercellular collagen. CD34-positive TCs in residual NMSs increased in number and formed most of the intrafusal components (Fig. 5F). In a case of inflammatory myopathy, a lymphocytic infiltrate was observed around some NMSs (Fig. 5G), and CD34-positive intrafusal TCs appeared in varying number (generally few).

## Discussion

Our results show the presence of TCs in NMSs. The stromal location in NMSs should therefore be added to the other sites in which TCs were recently identified as a new stromal cell type. The rationale for the terms used to name these cells (Popescu coined the term telocyte to prevent further confusion with other interstitial/stromal cells) and their characteristic components was established by the authors who described this new cell type [see 1, 2, 29].

Telocytes were mainly distinguished from other types of cells in NMSs by their distribution (location), relationship, morphology, immunohistochemical profile and ultrastructural characteristics (small body, presence of long, moniliform, convoluted, thin emerging Tps, alternating thin segments—podomeres—with dilations—podoms—and absence of basement membrane). TCs located in the inner and outermost layer of the capsule were CD34-positive and EMA-negative, whereas another cell type in the capsule (perineural cells) was EMA-positive and CD34-negative. Moreover, perineural cells did not present a moniliform aspect and were surrounded by a basement membrane. The intrafusal TCs were distinguished from: (a) Schwann cells, which were located around nerve fibres, showed S-100 positivity and were surrounded by a basement membrane, (b) pericytes, which were located around endothelial cells, with which they shared a basement membrane 30, 31 and were CD34-negative and α-SMA-positive, (c) endothelial cells, which adopted a typical arrangement and were positive for anti-CD34 and anti-CD31 (TCs were CD34-positive, but CD31-negative), and (d) intrafusal skeletal muscle cells, which were desmin-positive and morphologically clearly distinguished.

Neuromuscular spindle is an encapsulated proprioceptor, in which TCs form the innermost and (partially) the outermost layers of the NMS capsule, and the internal capsule. In the latter, TCs surround NMS intrafusal components, which comprise striated muscle fibres, nerves and blood vessels, adopting a special organization in these microanatomic structures. TCs have previously been identified in striated muscle, nerves and vessels found in several locations other than in NMSs. Thus, in rat, mouse and human skeletal muscle, TCs are arranged in a 3D network distribution of their Tps, which are in close vicinity to myocytes, satellite cells, capillaries and nerve endings 26. A normally occurring population within the endoneurium of peripheral nerves that is distinct from conventional fibroblasts and Schwann cells was also suggested from 1993 onwards 32, 33. Around blood vessels, TCs form a continuum from adventitia of the major vessels to the periphery of the microvasculature, especially small vessels 14, 15, 18, 26. In NMSs, all these components converge and the TCs are numerous, providing an ideal microanatomic structure for TC study.

Immunohistochemically, no single marker specific for TCs has been found 2 and immune labelling may be challenged 11. TCs in NMSs were positive for anti-CD34 and anti-vimentin, as occurs with TCs in other locations 2, 30, 34, 35. Considering that all the cells with ultrastructural characteristics typical of TCs in NMSs were positive for anti-CD34 and only occasionally for anti-CD117 (c-kit), our results coincide with the opinion that, although CD34 labelling does not permit unequivocal TC identification, it is still the best available immunohistochemical choice for this purpose 2. However, CD117 seems useful for the identification of cells with TC features 11, 23 and a different expression of c-kit is possible, depending on the method of inclusion and incubation. Furthermore, TCs express c-kit in certain circumstances and the expression of micro-RNA (*e.g*. miR-193), which regulates c-kit, differentiates TCs from other stromal cells 35. In any case, the precise identification of TCs is still achieved by electron microscopy.

Several roles for NMS TCs may be postulated: guide during NMS development, mechanical support with passive control of NMS activity and active involvement in the control of muscle tone and motor activity.

Telocytes may participate in NMS development. Together with the action of axon guidance molecules (*e.g*. semaphorin III and neurotrophin responsive cells – 36, 37), TCs and perineural cells may cooperate as conductors of the other NMS structures. Thus, in the region in which small primary sensory terminals establish neuromuscular contacts, the perineurium of the supplying nerve fascicles has traditionally been considered as extending and forming most of the external NMS capsule 38. According to our observations in developing NMSs, the pre-existing TCs (present around myotubes and associated microvasculature, and in the endoneurium of the supplying nerve fascicles) may have a similar role, increasing their number and forming a network that partially or totally surrounds each intrafusal structure, contributing to their interconnection.

Telocytes and perineural cells may provide mechanical support and, together with their special microenvironment, participate in the control of muscle tone and motor activity. Indeed, the Tp network delimits subcapsular and intrafusal spaces where there is a specific microenvironment, which shows an alcianophylic reaction characteristic of glycosaminoglycans 39. The presence of hyaluronic acid has been demonstrated in these spaces 40. Therefore, TCs and their microenvironment may act as a form of packaging around the intrafusal structures and contribute to mechanical stimuli and deformation of receptor terminals, which respond to changes in muscle length or tension. Mechanical stimuli lead to a modification in the permeability of the nerve membrane, depolarization of the ending and initiation of impulse activity, which is then propagated along the axon.

In NMSs, TCs may be actively involved in controlling muscle tone and motor activity. Indeed, TC location is strategic and the interconnected TCs form a continuous network, which could act in cell-to-cell signalling. Thus, a paracrine and/or juxtacrine intercellular modulation between TCs and cardiomyocytes has been proposed 7. In NMSs, homo- and heterocellular communications of TCs could therefore occur by means of shed vesicles and close apposition as has been suggested for TCs and cardiomyocytes 41. This role in NMSs requires further research, as for other TC locations 3, 4, 8, 11, 12, 18.

Telocytes have been involved in the pathogenesis of several pathological processes. They have, for example, been considered a common origin of both PEComas and GISTs 42 and related to amyloid deposits in human-isolated atrial amyloidosis 43. We have described a new type of choroid papilloma: ‘choroid plexus amyloidogenic elastopapilloma’ 44 and the TCs recently described in choroid plexus 25 could be involved in the lesion (L Díaz-Flores, R Gutérrez, FJ Sáez, L Díaz-Flores Jr., JF Madrid, unpublished observation). Although we have yet to obtain NMSs in pathological skeletal muscle specimens processed for electron microscopy, the changes in intrafusal CD34-positive cells under light microscopy suggest TC modifications in residual NMSs between infiltrative musculoaponeurotic aponeurosis and in NMSs present in inflammatory myopathy. Further research is also required in this field.
